# Railway Catenary Condition Monitoring: A Systematic Mapping of Recent Research

**DOI:** 10.3390/s24031023

**Published:** 2024-02-05

**Authors:** Shaoyao Chen, Gunnstein T. Frøseth, Stefano Derosa, Albert Lau, Anders Rönnquist

**Affiliations:** 1 Department of Structural Engineering, Norwegian University of Science and Technology (NTNU), 7491 Trondheim, Norway; 2 Department of Civil and Environmental Engineering, Norwegian University of Science and Technology (NTNU), 7491 Trondheim, Norway

**Keywords:** systematic mapping, condition monitoring, railway catenary system, monitoring targets, sensor types, monitoring platforms

## Abstract

In this paper, a different approach to the traditional literature review—literature systematic mapping—is adopted to summarize the progress in the recent research on railway catenary system condition monitoring in terms of aspects such as sensor categories, monitoring targets, and so forth. Importantly, the deep interconnections among these aspects are also investigated through systematic mapping. In addition, the authorship and publication trends are also examined. Compared to a traditional literature review, the literature mapping approach focuses less on the technical details of the research but reflects the research trends, and focuses in a specific field by visualizing them with the help of different plots and figures, which makes it more visually direct and comprehensible than the traditional literature review approach.

## 1. Introduction

In an electrified railway system, the locomotive and multiple units acquire electric current via a pantograph in the catenary system, which is a vital component ensuring the efficient operability and reliability of the entire railway system [1]. Any failures or defects in the catenary system can lead to significant delays or safety hazards [2]. Therefore, researchers have been focusing on condition monitoring for the catenary system to enhance its operability and reliability. As a result, a considerable amount of research has been conducted in this area over the past few decades. This paper presents a systematic mapping of recent research on catenary monitoring, which aims to provide a structured overview of the type of research and results in this field by categorizing and visualizing them in the form of a map [3]. The primary objective of this work is to establish the current status and specific trends in research on condition monitoring of railway catenary systems, thereby guiding new research efforts in the most efficient manner.

In the realm of catenary monitoring, various facets can be taken into consideration. Firstly, monitoring targets can encompass different types, including the contact force between the catenary and pantograph, arcing, and the catenary components such as insulators and droppers, among others. Secondly, a diverse range of sensors are employed in the monitoring process, such as cameras, accelerometers, and so on. Lastly, the platform used to install those sensors may vary, ranging from normal-vehicle-based, dedicated-vehicle-based, to non-vehicle-based platforms.

The existence of multiple facets may introduce difficulties in structuring and extracting essential information in the review. However, the systematic mapping approach proves to be effective in addressing these challenges. By representing different facets on different axes of a map, systematic mapping can successfully integrate and summarize various aspects within a research field. This allows for the unveiling of the interrelationships between these different facets. Consequently, with the information expressed in various visual maps, systematic mapping provides a clear and comprehensive overview of a specific research field, shedding light on the intricate correlations between its different facets. The results of the mapping process are presented in Section 4 of this paper. The obtained mapping results provide a comprehensive understanding of the prevailing sensors utilized for monitoring specific types of defects, discerning the current trends in monitoring technology, and identifying prominent research groups in this field, along with their collaborative endeavors.

It is important to note that the literature systematic mapping technique utilized in this research differs from the conventional literature review. Since maps offer an effective and concise way to present large-scale information, literature systematic mapping provides a more structured and coarse-grained overview, in comparison to systematic reviews [3], with less emphasis on details in the literature but more on the overall structure. The methodology presented in the following section provides a comprehensive understanding of what systematic mapping entails.

## 2. Methodology

**Figure 1:**
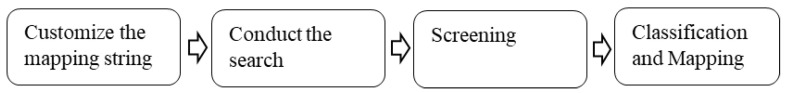
The process of systematic mapping.

The systematic mapping process encompasses several distinct stages. Initially, a meticulously tailored search string is formulated and employed to conduct an extensive literature search across pertinent databases. Subsequently, a screening procedure is executed to eliminate irrelevant literature that fails to satisfy the research criteria. This step assumes significant importance, as the database search results may encompass literature from disparate fields and subjects, as well as duplicates of identical works. Following this, a comprehensive classification process is undertaken to identify and categorize the various dimensions of the research literature pertaining to catenary monitoring. This classification process serves as a fundamental pillar for the systematic mapping, enabling effective visualization of these dimensions and efficient extraction of the underlying information. A detailed explanation of the systematic mapping methodology can be found in references [3,4,5] while an overview of the process is depicted in Figure 1.

The subprocesses presented in Figure 1 are described in more detail in the subsections below.

### 2.1. Establishing the Search String

**Figure 2:**
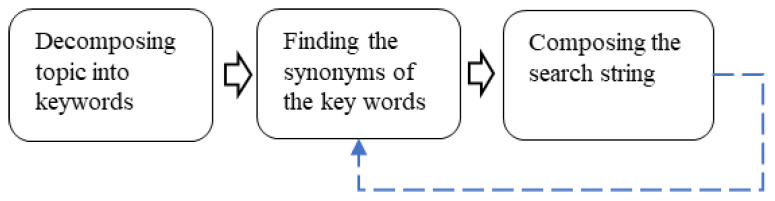
The workflow of composing the search string.

The search string was established using the workflow shown in Figure 2.

The overall topic and focus of the systematic mapping were formulated into a single sentence: “condition monitoring of the railway catenary system”. This sentence was then broken down into its main keywords: “condition monitoring”, “catenary”, and “railway”. Then, relevant synonyms of these keywords were determined by considering dictionaries and the authors’ initial knowledge of the field. The process of finding synonyms was iterative, as indicated in Figure 2, as new synonyms arose during the research process. The keywords and final list of corresponding synonyms are shown in Table 1 below.

These keywords were then combined to construct a final string of search terms for the systematic literature review:

*(“catenary” OR “overhead contact line” OR “contact line” OR “contact wire”) AND (“condition monitoring” OR “monitoring” OR (((“anomaly”) OR (“faults”) OR (“defects”)) AND ((“diagnosis”) OR (“detection”) OR (“monitoring”)))) and (“railway” or “rail”)*.

### 2.2. Conducting the Search

To ensure comprehensive coverage of the literature, five widely used and topic-related databases were selected: ScienceDirect, Web of Science, Engineering Village, Scopus, and IEEE. Journal articles and conference papers were included in the literature search. Moreover, the search had no start-year limit, so literature was sought from as far back as possible. The literature search was conducted in March 2023, and the search results and corresponding databases are presented in Table 2.

The search resulted in a total of 1417 references distributed across the different databases.

### 2.3. Screening the Search Results

After conducting the search, the next step was to screen the results. First, any duplicates that appeared due to overlapping literature across the different databases were removed. This reduced the number of references from 1417 to 154.

The criteria listed below were used for further screening:

- Written in English.
- Full text available online.
- The content should be directly about the condition monitoring of the catenary system.

The first two criteria are straightforward, but the third one requires closer exploration. In this study, the abstract was primarily used to assess the relevance of each paper. If the content was not clearly stated in the abstract, the introduction or conclusion section were also consulted. If necessary, the entire paper was reviewed to determine its suitability for inclusion. Following the screening process, a total of 154 papers were found that met the inclusion criteria.

### 2.4. Classification and Mapping Scheme

The objective of this paper is to establish the status of and current trends in condition monitoring in the railway catenary system by analyzing the available literature on this topic. The literature contained in the search results above contains the information required to reach the objective.

*Condition monitoring in the railway catenary system* refers to the process of observing any response or feature of the railway catenary system for the purpose of determining the condition of the system. The topic is, therefore, very broad and general, and it is difficult to extract objective information from the literature with this definition alone.

There are many specific examples of condition monitoring:

**-**  Contact force measurement for determining contact wire irregularity.
**-**  Image acquisition with an area-scanning camera to identif dropper defects.
**-**  Acceleration measurement for finding the catenary tension force.

Which breaks down into a combination of two categories:

**-**  *What* is the target/objective of monitoring? (e.g., contact wire irregularity)
**-**  *How* is it monitored? (e.g., contact force measurement)

In order to establish the status of and current trends in condition monitoring of the railway catenary system, it is therefore necessary to extract and quantify the *what* and *how* from the literature contained within the search results.

Eventually, after an iterative process which involved analyzing the literature and adding/removing facets, the following three facets were found to concisely describe the *what* and *how* of condition monitoring in the railway catenary system contained within the literature: *monitoring targets, sensor types and monitoring platform*, see Table 3.

A more detailed description and definition of the different elements within each facet are given in the following chapter.

In addition to the *what* and *how* described by facets above, the *when* and *who* is also readily available in the search results and will be further analyzed in the following section to establish the status of and current trends in condition monitoring of the railway catenary system.

## 3. Results and Discussions

### 3.1. Overview of the Literature

**Figure 3:**
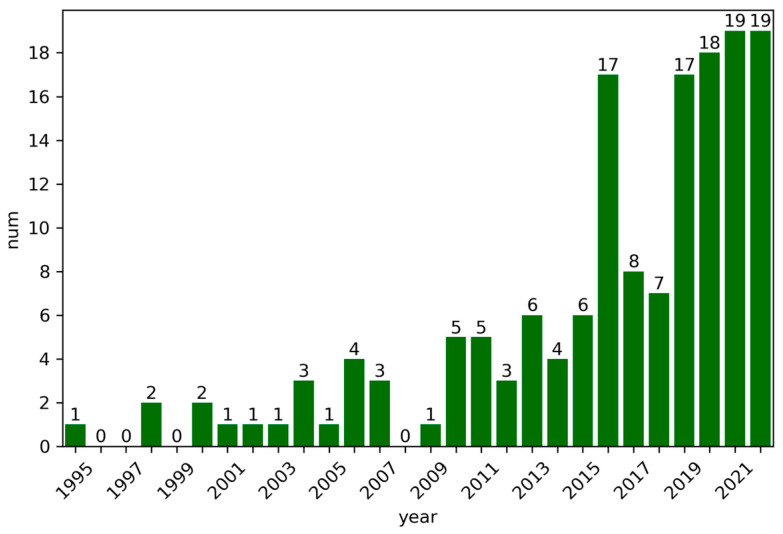
The publication number per year.

Before presenting the mapping results, it is informative to examine the general research trends in the field of railway catenary condition monitoring. Figure 3 displays the number of publications related to railway catenary system monitoring over time. The first publication found to be on railway catenary system monitoring was from 1995 [6], and from 1995 to 2015, the number of publications steadily increased. In 2015, there were only six publications on this topic. However, in 2016, the number of publications increased dramatically to seventeen, followed by a decrease to eight and seven in the following two years. In 2019, the number of publications experienced another surge, reaching 17 and then gradually increasing to 19 in 2022. Therefore, it can be inferred that, since 2015, there has been a sudden increase in publications on railway catenary system monitoring. There could be multiple reasons for this phenomenon. One possible reason is the recent advancements in artificial intelligence, which have yielded significant progress in condition monitoring, drawing more attention to this field and leading researchers to explore the application of these techniques in railway catenary system monitoring. Additionally, the rapid development of high-speed railways worldwide and the need for efficient and intelligent monitoring and maintenance techniques for large-scale railway networks may have also driven the research on catenary system monitoring forwards at a fast pace.

**Figure 4:**
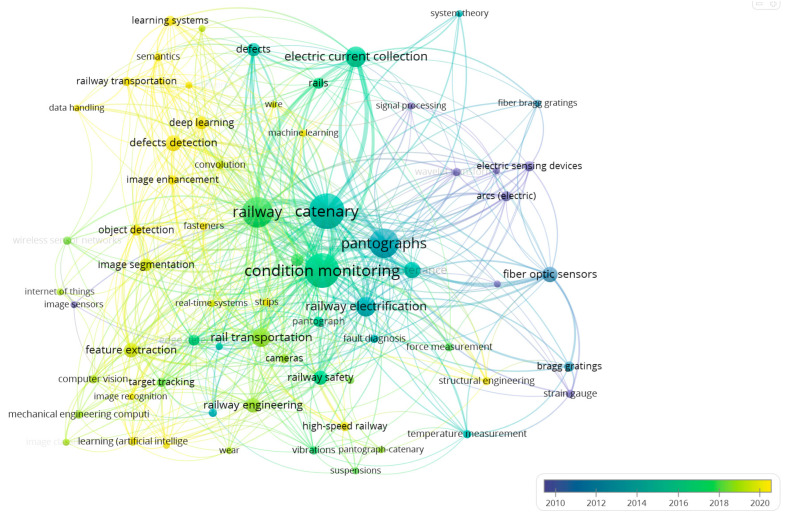
The keywords’ relationships across all the articles.

To provide a comprehensive overview of the literature on railway catenary system monitoring, it is useful to visualize the keywords that have appeared in these studies. Figure 4 presents a visual representation of these keywords, where the size of the marker and the visualization of the texts indicates the frequency of the occurrence of a particular keyword, the lines indicates certain connections between two keywords, and the thickness of the lines represents the frequency of these two words appearing in the same paper [7]. The color saturation reflects the mean year of a specific key word appears. In Figure 4, we have included only those keywords that appeared at least three times in all the articles, resulting in a total of 68 words. These keywords provide insights into the content and focus of the literature on catenary system monitoring. Moreover, the latest keywords are indicative of the more recent monitoring techniques and targets in catenary monitoring. For instance, *convolution*, *learning systems*, and *image enhancement* are related to deep learning and computer vision, which will be discussed in more detail in the following section.

**Figure 5:**
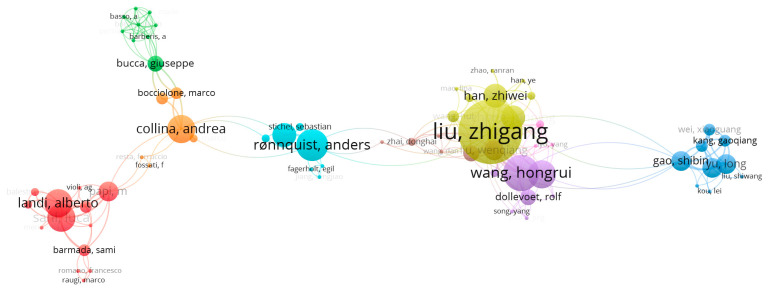
The authorship of all the articles.

Furthermore, a visual overview of authorship is presented, similarly to Figure 4, in Figure 5. However, in Figure 5, color saturation is not utilized as it would obscure the representation of different clusters, which denote cooperation between different authors. These clusters provide information about the researchers and collaboration between them; however the same color does not necessarily indicate the same institution, but instead shows close cooperation. Figure 5 does not include all the authors with publications on catenary monitoring but includes those with the most publications in this field in recent years. The corresponding institutions are Southwest Jiaotong University (SWJTU), Delft University (TU Delft), the Norwegian University of Science and Technology (NTNU), and the Politecnico University of Milano (Polimi). As evident from the figure, several leading institutions have established close cooperation with each other. For instance, SWJTU collaborates with TU Delft and NTNU. Notably, SWJTU has the highest number of collaborations with other institutions. Additionally, Zhigang Liu, affiliated with SWJTU, is the author with the highest number of publications. Liu and his colleagues have made significant contributions to the field of catenary monitoring through their work on visually inspecting the catenary system components using computer vision techniques, such as the catenary support device monitoring [8,9,10]. NTNU has contributed to the field of catenary condition monitoring by utilizing wayside photogrammetry methods, such as dynamic response analysis [11,12]. TU Delft has contributed to this field by utilizing entropy-based methods and analyzing the wavelength of the pan-head acceleration [13,14]. Polimi also focuses on utilizing the pan-head acceleration to detect the catenary defects [15,16]. The following part of this section provides further insights into their work.

### 3.2. Monitoring Targets

The classification and mapping processes which are described in Section 3 identified *monitoring targets* as one of the facets in condition monitoring of railway catenary systems. The monitoring targets are presented in Table 3 and include everything that is part of the catenary system, including “contact point” and “contact force”, although these targets are on the boundary between the catenary system and the pantograph system. Only monitoring targets which occurred in more than two references are included in their own category, other monitoring targets, such as the bird nest [17] and catenary guide height [18], have been categorized as “other monitoring targets”. The classification resulted in 10 terms (except the “other monitoring targets”), as shown in Table 4.

Table 4 provides detailed explanations of these 10 terms. To facilitate the readability of the mapping plots, we have used simplified names for each term. For example, “supportive components” refers to catenary supportive components, such as insulators, brace sleeves, double sleeve connectors, and so on.

**Figure 6:**
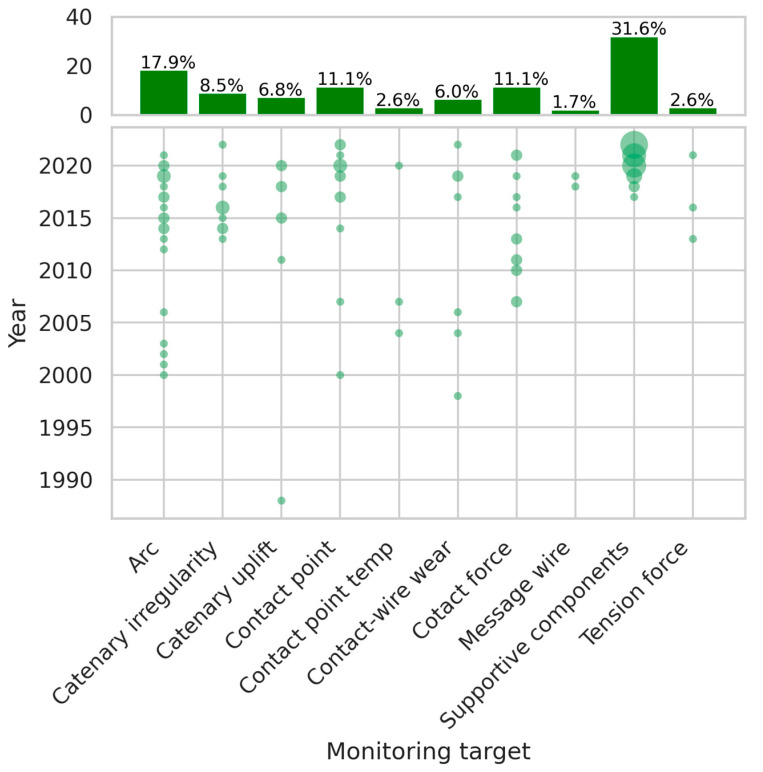
The proportion of the top ten monitoring targets and yearly variation.

Figure 6 is a combination of a histogram for each of the ten predetermined targets and a corresponding scatter plot across the years. The scatter plot provides information about the distribution of articles across different monitoring targets over time. The size of the round spot is proportional to the number of articles on each target. The histograms present these monitoring targets and their relative frequency of appearance in the literature as a percentage. It is noteworthy that Figure 6 exclusively focuses on the aforementioned ten monitoring targets, as otherwise, “other monitoring targets”, with its considerable volume, would compress the proportions of the ten categories, thus attenuating the distinction in their relative associations. Therefore, “other monitoring targets” are excluded from the calculation and are not plotted in Figure 6. Additionally, in the histogram, the percentages were calculated by dividing the number of articles that focused on each monitoring target by the total number of articles that focused on the ten predetermined targets. However, “other monitoring targets” are depicted in Figure 9 in Section 3.5.

The histogram in Figure 6 illustrates the occurrence of different monitoring targets, with “catenary supportive components” being the most studied target at 31.6%, followed by the arc at 17.9%. The contact point and contact force also received considerable attention at 11.1% each. It should be noted that these percentages were calculated based on the number of articles that focused on these ten monitoring targets alone, and not on any other targets outside of this group. Further examination of the scatter plot in Figure 6 reveals that the interest in studying catenary supportive components has increased significantly in recent years. In 2017, Tang and Jin proposed a framework for segmenting catenary poles and gantries [19], while in 2018, Liu et al. applied a deep convolutional neural network to detect defects in catenary supportive components [20]. These two papers were among the earliest works in this area, and since then, the number of articles focusing on this target has increased rapidly, with 11 such papers published in 2022. Most of these articles are from Southwest Jiaotong University, and focus on inspecting the catenary system of the Chinese high-speed railway [10,21,22,23,24,25,26,27,28,29,30,31,32,33,34,35,36,37,38,39,40,41,42]. The monitoring is achieved using high-definition cameras installed on dedicated inspection vehicles, with image capturing primarily taking place at night to avoid the interference of complex daytime backgrounds. It is worth noting that, while early papers focused on the feasibility of using deep learning-based computer vision to monitor catenary supportive components [20,21], recent works, especially in 2022, have focused more on improving the precision and performance of these approaches for specific components. For example, papers [8,39] have adopted advanced methods to improve the accuracy of insulator defect detection.

Following “catenary supportive components”, “arc detection” accounts for the second-largest proportion. Further examination of Figure 6 reveals that the research interest in arc detection has been evenly distributed over the years, with a gap from 2007 to 2011. Before 2007, arc detection mainly relied on sensors such as phototubes [43,44,45,46] and infrared cameras [47]. From 2012 to 2016, several conference papers focused on arc detection, with most of them being based on digital cameras [48,49,50,51,52,53,54,55]. These studies mainly apply classical and traditional algorithms instead of deep learning-based computer vision algorithms. Most of these papers are authored by Aydin Firat University, Turkey. However, in research published in and after 2017 [56,57,58,59,60,61,62,63,64], researchers began to use camera sensors and deep learning-based computer vision techniques. “Contact point” and “contact force” account for 11.1% of the total amount, after “supportive components” and “arc”. Both of these monitoring targets have a relatively even distribution throughout the years, as shown in Figure 6. One reason for “arc”, “contact point”, and “contact force” accounting for relatively large proportions compared to other monitoring targets might be that they can more directly reflect the health condition of the catenary and pantograph compared to other monitoring targets.

### 3.3. Sensor Types

In addition to the monitoring targets, the classification and mapping process also identified *sensor types* as one of the key facets in describing condition monitoring of railway catenary systems. Similar to defining the monitoring targets in Section 3.2, these seven terms do not encompass all the sensor types utilized in the catenary monitoring domain, but rather the most prevalent ones. A discussion about the other sensor types will be presented in Section 3.5.

**Figure 7:**
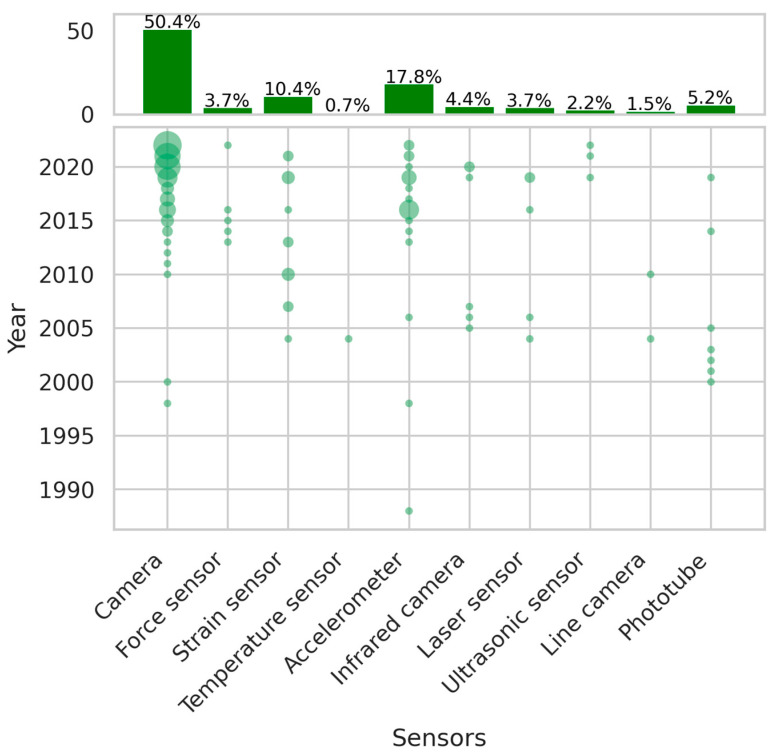
The proportion of the top ten sensors and yearly variation.

The histogram in Figure 7 illustrates the relative proportions of the sensor types used for monitoring the condition of railway catenary systems, as identified in Table 5. As with the histogram in Figure 6, the percentage calculations presented in Figure 7 exclude sensor types outside the ten listed in Table 5. Additionally, the scatter plot in Figure 7 depicts the yearly variation in the use of these sensor types. Notably, camera sensors account for a large number [8,64,65,66,67,68,69,70,71,72,73,74,75,76,77,78,79,80,81,82,83,84,85,86,87]. However, the distribution of camera sensors through the years is not even. Before 2000, camera sensors were already being utilized to monitor catenary systems [86,87], and traditional and simple vision techniques were employed to analyze the images. From 2000 to 2010, camera sensors faced a gap in their usage. However, since 2010, they have regained research interest, and this period can be reasonably divided into two sub-periods. The first sub-period utilizes camera sensors with traditional algorithms, such as Canny edge detection, while the second sub-period involves the use of camera sensors with deep-learning-based computer vision techniques for monitoring. Although there is no definitive dividing line between these two sub-periods since the first sub-period gradually evolves into the second, we can take 2017 as the line of division, given that the first paper jointly using camera sensors and deep-learning-based computer vision techniques for monitoring was published in that year [56]. Since then, this type of paper has gradually become the dominant type in this field. Among these papers, Southwest Jiaotong University has contributed the most in terms of utilizing camera sensors and deep-learning-based computer vision algorithms for detecting defects in the catenary system’s supportive components, as mentioned in Section 4.2.

The accelerometer is the second most popular sensor type, contributing 18.2%, and it has shown a relatively even distribution through the years from 1989 to 2022 [88,89,90,91,92,93,94,95,96,97,98,99,100,101]. Most studies use accelerometers to obtain the pan-head acceleration, which can determine the catenary’s health condition through certain algorithms, such as those presented in [13,15]. Additionally, accelerometers can also be installed on the contact wire or the message wire to measure vertical acceleration, enabling the evaluation of wave propagation and dynamic behavior in the catenary system [89,91,94,95,96,97]. Strain sensors are the third most popular sensor type, accounting for 10.6% of all sensors used [102,103,104,105,106,107]. In these studies, the majority of accelerometers and strain sensors are FBG-based (Fiber Bragg Grating-based) sensors. The advantage of FBG-based sensors is that they are not influenced by electromagnetic interference, making them an ideal sensor type for monitoring catenary and pantograph-related issues, where electromagnetic interference is a significant issue for sensors.

### 3.4. Monitoring Platforms

**Figure 8:**
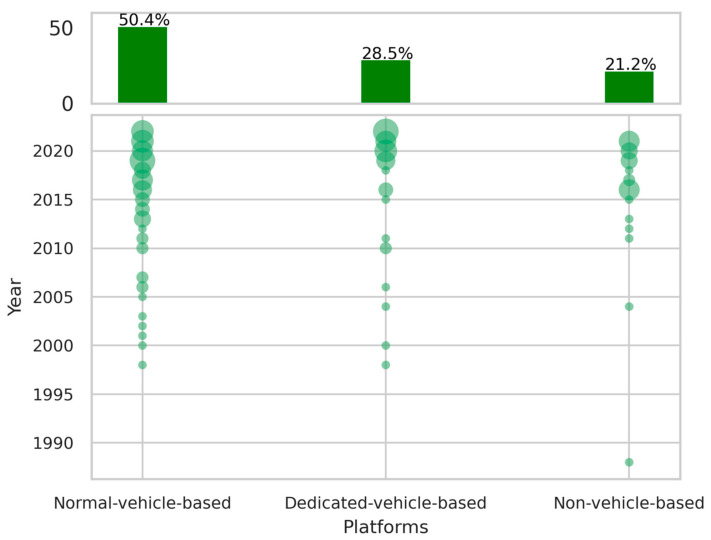
The proportion of platforms.

The variation in monitoring platforms used through the years is the final facet in the condition monitoring of railway catenary systems as shown in Table 6, and answers the question of *how* it is performed together with the sensor types. The monitoring platform refers to the platform on which the monitoring sensors are installed. As illustrated in the histogram in Figure 8, the normal-train-based platform has the highest proportion (50.4%) compared to the dedicated-train-based platform (28.5%) and non-vehicle-based platform (21.2%). The scatter plot in Figure 8 shows that the yearly distribution of these three platforms is relatively even, and overall, research on condition monitoring of railway catenary systems is active on each of the three monitoring platforms. However, the dedicated-train platform has shown significant growth in recent years, which can be attributed to the research efforts from SWJTU, as mentioned in Section 3.3

### 3.5. Relationship between Platforms, Sensor Types, and Monitoring Targets

**Figure 9:**
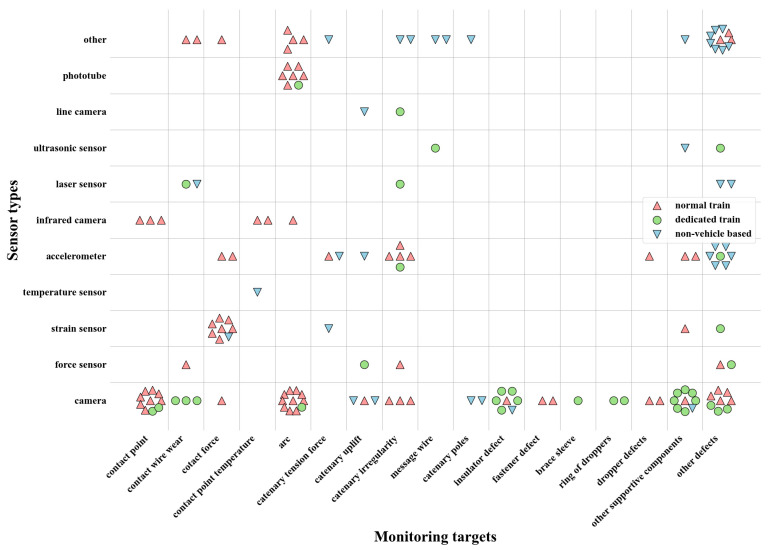
Plot showing the relationship among monitoring targets, sensor types, and platforms.

In addition to examining each facet of monitoring targets, sensor types, and platforms individually, in Section 3.2, Section 3.3 and Section 3.4, it is also important to explore the relationships among these three facets. Figure 9 provides an overview of the relationships among different sensors, monitoring targets, and platforms. The vertical axis of the plot represents different sensor types, while the horizontal axis represents different monitoring targets. The different colored shapes in the plot represent different platforms used in catenary monitoring research. Each colored shape represents a paper that focuses on catenary monitoring. Moreover, Figure 9 further refines the monitoring targets along the horizontal dimension. As depicted in Figure 7, there is a broad category called “supportive components”, which is further classified into specific components such as catenary poles, insulator defects, fastener defects, brace sleeves, rings of droppers, dropper defects, and other supportive defects. These are the most common supportive components monitored, while the less commonly monitored supportive components are grouped under the category of “other supportive defects”. In addition, as mentioned in Section 3.2, there is a category titled “other monitoring targets”; it was not included in the calculations in Section 3.2, but in this section, it is being used and it is plotted in Figure 9. It should be noted that the total number of points in Figure 9 does not correspond to the total number of papers reviewed in this research, as some aspects of the papers were neglected for the sake of brevity and representativeness.

In examining Figure 9, it is clear that the camera sensor is the most prevalent type of sensor and is widely used for a variety of monitoring targets, including contact point detection, arc detection, and catenary supportive component defect detection, such as insulator defects. With recent advancements in computer vision techniques, cameras are capable of handling nearly all types of monitoring targets. Normal and dedicated train platforms are the most common platforms used to install camera sensors, rather than wayside platforms. In addition to cameras, accelerometers are also widely used for monitoring catenary irregularity, and can be used on either train or wayside platforms. When considering the vertical axis in Figure 9, it becomes evident that multiple sensors are utilized for arc detection and that train-based platforms are used for this purpose. Conversely, camera sensors are primarily used to monitor catenary supportive components and are mainly deployed on dedicated train platforms.

## 4. Conclusions and Future Work

### 4.1. Conclusions

The present study aimed to map the literature related to the monitoring of railway catenary systems, exploring three facets: monitoring targets, sensor types, and monitoring platforms. Independent analyses were conducted for each facet, yielding the following findings:

- Research on condition monitoring of railway catenary system has increased significantly since 2017.
- Key research groups and researchers have been identified in the field of condition monitoring of railway catenary systems. Several of the research groups have already established collaboration.
- Monitoring of catenary supportive components, such as insulators, brace sleeves, and double sleeve connectors, has become increasingly popular in recent years and is the dominant monitoring target in current research.
- Camera sensors dominate the other types of sensors by a significant margin, and their application in condition monitoring of railway catenary system is still increasing year by year.
- Monitoring based on normal trains is the most common monitoring platform in condition monitoring of the railway catenary system, but the dedicated-train-based and non-vehicle-based platforms are also commonly used in condition monitoring of railway catenary systems and these three monitoring platforms still remain active research fields.
- The popularity of camera sensors for railway catenary monitoring may be attributed to the versatility of the camera sensor in many monitoring tasks and the advancements in artificial intelligence and the maturity of deep-learning-based algorithms.

### 4.2. Future Work

The current research on catenary system condition monitoring presents certain limitations. Although cameras have significantly advanced various monitoring tasks, they typically require installation on specialized inspection trains. This allows them to capture images at low speeds and during the nighttime, ensuring clear photos against a dark background, which aids in target recognition and identification. A promising research direction involves mounting these cameras on regularly operated trains and developing computer vision techniques to process images with complex backgrounds and potentially lower quality. Additionally, despite the growing interest in computer vision-based monitoring, there is still a need to develop techniques that utilize time-series signals, such as acceleration and contact force between the contact wire and the pan-head. Force sensors and accelerometers are more accessible and cost-effective. Developing monitoring techniques that effectively utilize force or acceleration signals would be a valuable research avenue.

## Figures and Tables

**Table 1:** Keywords and corresponding synonyms in final search string.

Keyword 1	Keyword 2	Keyword 3
catenary	condition monitoring	railway
overhead contact line	monitoring	rail
contact wire	anomaly diagnosis	
overhead line	anomaly detection	
	fault(s) detection	
	fault(s) diagnosis	
	defect(s) detection	

**Table 2:** The search results obtained from the relevant databases in March 2023.

Database	Search Results
ScienceDirect	442
Web of Science	177
Engineering Village	280
Scopus	328
IEEE	190

**Table 3:** Different facets describing condition monitoring of railway catenary systems.

Facet 1 Monitoring Targets	Facet 2 Sensor Types	Facet 3 Monitoring Platform
arc	camera	normal train
catenary irregularity	force sensor	dedicated train
catenary uplift	strain sensor	non-vehicle based
contact point	temperature sensor	
contact point temp	accelerometer	
contact wire wear	infrared camera	
contact force	phototube	
message wire		
supportive components		
tension force		

**Table 4:** Different terms under monitoring targets.

Facet 1 Monitoring Targets	Description
arc	Monitoring/detection of electric arcing between the pantograph and catenary
catenary geometry	Monitoring/detection of the catenary geometry irregularity such as the irregularity of stagger
catenary uplift	Monitoring/detection of contact wire uplift from static equilibrium
contact point	Localization of contact point and/or detection of contact between contact wire and pantograph
contact point temp	Monitoring/detection of temperature of the contact point
contact wire wear	Monitoring/detection the level of wear of the contact wire
contact force	Monitoring/detection the contact force between pantograph and catenary
message wire	Monitoring/detection the damage on the message wire
supportive components	Monitoring/detection of damage on the catenary support components, i.e., insulators, brace sleeves, and double sleeve connectors.
tension force	Monitoring/detection of the tension force in the contact wire

**Table 5:** Different terms under sensor types.

Facet 2 Sensor Types	Description
Camera	Digital or analog cameras that capture images or image processing
Force sensor	Sensors measuring force, e.g., a load cell based on strain-gauges or fiber bragg grating (FBG)
Strain sensor	Sensors measuring strain, e.g., electrical resistance strain gauge or optical FBG sensors
Temperature sensor	Sensors measuring temperature, e.g., RTD, thermocouple or optical FBG sensors
Accelerometer	Sensors measuring acceleration
Infrared camera	Camera which produce images from infrared (IR) radiation of objects
Laser sensor	Sensor that use laser technology to detect or measure certain parameters or conditions
Ultrasonic sensor	Sensor that utilizes ultrasonic wave to realize the defect detection for metal components
Line camera	Camera captures a single line of pixels at a time when there is relative movement between object and camera
Phototube	Sensor that produces a signal proportional to light intensity

**Table 6:** Different monitoring platforms.

Facet 3 Monitoring Platform	Description
normal train	Sensors are installed on the normally operated train, such as a passenger train [65]
dedicated train	Sensors are installed on the dedicated train, such as an inspection train [8]
non-vehicle based	Sensors are not installed on the train, but on the wayside, such as on the catenary supportive system [11,95]
